# Prognostic Power of the Naples Score in Non-Small Cell Lung Cancer: Can Inflammation and Nutrition Predict Survival?

**DOI:** 10.3390/jcm14113715

**Published:** 2025-05-26

**Authors:** Pınar Peker, Aslı Geçgel, Alpay Düşgün, Oğuzcan Özkan, Berna Bozkurt Duman

**Affiliations:** 1 Department of Medical Oncology, Adana City Hospital, Adana 01230, Turkey; alpaydusgun@hotmail.com (A.D.); berboz@hotmail.com (B.B.D.); 2 Department of Medical Oncology, Ege University Faculty of Medicine, Izmir 35040, Turkey; dr.asltrgt@gmail.com (A.G.); droguzcanozkan@yahoo.com (O.Ö.)

**Keywords:** non-small cell lung cancer, Naples prognostic score, inflammation, nutrition, survival

## Abstract

**Objectives**: This study aimed to investigate the prognostic value of the Naples Prognostic Score (NPS), a composite index of inflammation and nutrition markers, in patients with non-small cell lung cancer (NSCLC) and to assess its role in predicting survival across clinical subgroups. **Methods**: A retrospective analysis was conducted on 250 patients diagnosed with NSCLC between 2018 and 2023. Patients were categorized into low (≤2) and high (>2) NPS groups based on the scoring system derived from neutrophil–lymphocyte ratio (NLR), lymphocyte–monocyte ratio (LMR), serum albumin, and total cholesterol levels. Survival outcomes were analyzed using Kaplan–Meier curves, log-rank tests, and univariate and multivariate Cox regression analyses. Receiver operating characteristic (ROC) analysis was performed to determine the discriminatory ability of NPS. **Results**: Patients with high NPS (>2) had significantly lower overall survival (median OS: 10.4 vs. 18.2 months, *p* < 0.001) and progression-free survival (median PFS: 7.3 vs. 12.5 months, *p* < 0.001) than those with low NPS. High NPS was found to be an independent prognostic factor in multivariate Cox regression analysis (HR: 1.98, 95% CI: 1.42–2.76, *p* < 0.001). ROC analysis showed an AUC of 0.78 for NPS in predicting survival. Subgroup analyses demonstrated the consistent prognostic impact of high NPS across histological subtypes, TNM stages, smoking status, albumin levels, and age groups. **Conclusions**: NPS is an independent and practical prognostic tool in NSCLC. Its use may enhance risk stratification and support personalized treatment planning, particularly in advanced-stage patients.

## 1. Introduction

Non-small cell lung cancer (NSCLC) is a heterogeneous group of diseases that constitutes approximately 85% of all lung cancers [1]. NSCLC is divided into subgroups based on histological types, such as adenocarcinoma, squamous cell carcinoma, and large cell carcinoma. The prognosis of patients diagnosed with NSCLC varies depending on several factors, such as the TNM stage, histopathological subtype, molecular features of the disease, and treatment options. Numerous factors, including age, stage, weight loss, lymph node, and pleural involvement, have been found to impact survival in studies involving patients with NSCLC [2]. Recent studies have highlighted the significant role of inflammation and nutritional status in cancer prognosis. Additionally, among patients with NSCLC, nutritional status was recently identified as a potential novel prognostic predictor for survival and a predictive marker for treatment-related toxicities [3].

In cancer patients, the inflammatory response and nutritional status play a decisive role in disease progression, treatment response, and overall survival [4]. Recent research has started to identify the molecular pathways that connect cancer and inflammation. Smoldering inflammation in the tumor microenvironment promotes angiogenesis, metastasis, the growth and survival of cancerous cells, the disruption of adaptive immunity, and a diminished reaction to hormones and chemotherapy. Recent findings imply that the creation of genomic instability by inflammatory mediators, which results in the accumulation of random genetic changes in cancer cells, is another mechanism behind cancer-related inflammation [5]. Researchers are also interested in the survival study of malignant tumors because of nutritional status as a host-related factor [6]. The systemic inflammatory response produced by peripheral immune cells can indirectly reflect the severity of local malignancy. Peripheral blood neutrophils, lymphocytes, monocytes, and platelets are widely used as systemic inflammatory markers to predict tumor prognosis and evaluate therapeutic response [7]. Chronic inflammation plays a pivotal role in cancer development by influencing key processes such as tumor proliferation, invasion, and metastasis through modulation of the immune response within the tumor microenvironment. It has been established that several molecular processes mediate the widespread connections between immune cells invading tumors and cancer cells [8].

At the same time, inflammation can be associated with malnutrition and can trigger negative conditions, such as cancer-related cachexia and weight loss. For this reason, inflammatory markers and nutritional indices have increasingly started to be used to predict cancer prognosis [9]. The Naples Prognostic Score (NPS) is a novel scoring system that integrates nutritional and inflammatory parameters to predict prognostic outcomes in various cancers. It comprises serum albumin, total cholesterol concentration, neutrophil-to-lymphocyte ratio (NLR), and lymphocyte-to-monocyte ratio (LMR), effectively reflecting a patient’s immune and nutritional status. Furthermore, compared to other scoring systems related to inflammation and nutrition, the prediction value of NPS is more accurate [10]. Elevated NLR and reduced LMR indicate a heightened systemic inflammatory state, which has been associated with immunosuppression and tumor-promoting microenvironments. Low albumin and cholesterol levels, on the other hand, reflect malnutrition and poor metabolic reserve, which impair tissue repair, immune competence, and treatment tolerance. Together, these components offer a comprehensive overview of the host–tumor interaction, thereby allowing NPS to serve as a robust predictor of disease progression and overall survival across various cancer types [11]. Studies have demonstrated the prognostic significance of NPS in malignancies such as gastric cancer, colorectal cancer, and hepatocellular carcinoma [12,13]. Additionally, its prognostic value has been investigated in other tumor types, such as esophageal, biliary tract, duodenal ampulla, and pancreatic cancer [14,15,16]. Prognostic scores based on inflammation and nutrition (NLR, PLR, PNI, and mGPS) have been linked to survival in NSCLC patients [17,18]. However, the role of NPS in predicting prognosis in patients with NSCLC has not been sufficiently studied.

Crucially, a large number of previous studies that assessed prognostic indices in NSCLC had small sample sizes, which limits how broadly the results can be applied. A few published studies, for example, used cohorts of fewer than 200–300 patients. The prognostic significance of oxidative stress scores and systemic inflammatory markers in early-stage lung adenocarcinoma, on the other hand, has recently been validated by larger, more reliable studies. Systemic inflammation-based classification was shown to be a significant predictor of prognosis in stage I lung adenocarcinoma in one study with 913 patients [19]. The ability of a novel oxidative stress score to predict survival in early-stage non-small cell lung cancer was also validated by a cohort of 955 patients [20]. These extensive investigations offer a more solid basis for assessing prognostic instruments in clinical oncology.

The aim of this study is to determine the prognostic significance of NPS in patients with NSCLC and to evaluate its survival predictability. In addition, we aim to examine the relationship between the prognostic impact of NPS in NSCLC patients and factors such as histological subtypes, TNM staging, smoking status, and nutritional level through subgroup analyses. In this way, it is believed that new prognostic models focusing on inflammation and nutritional status can contribute to personalized treatment approaches for NSCLC patients.

## 2. Materials and Methods

This retrospective study included 250 patients diagnosed with non-small cell lung cancer (NSCLC) between 2018 and 2023 who had a minimum follow-up duration of six months. Demographic data, laboratory parameters, and treatment modalities were retrospectively collected and analyzed using the hospital’s electronic medical record system. The Naples Prognostic Score (NPS) was calculated based on four parameters: neutrophil-to-lymphocyte ratio (NLR) > 3, lymphocyte-to-monocyte ratio (LMR) < 4, serum albumin < 3.5 g/dL, and total cholesterol < 180 mg/dL. Each abnormal parameter was scored as one point, resulting in a total score ranging from 0 to 4. Patients were classified into two groups according to their NPS: low (≤2) and high (>2). Subgroup analyses were carried out to assess the prognostic impact of NPS across different clinical characteristics, including histological subtypes (adenocarcinoma and squamous cell carcinoma), TNM stage (early stage I–II vs. advanced stage III–IV), smoking status (never, former, current smokers), serum albumin levels, and age groups (≤65 vs. >65 years). TNM staging was performed according to the 8th edition of the American Joint Committee on Cancer (AJCC) staging system. The association of NPS with survival was examined within each subgroup to evaluate its independent prognostic relevance. All data were anonymized prior to analysis. Ethical approval for the study was obtained from the institutional review board, and the requirement for informed consent was waived due to the retrospective nature of the study.

Statistical analyses were performed using IBM SPSS Statistics version 26.0. A *p*-value of <0.05 was considered statistically significant. Descriptive statistics were used to summarize clinical and demographic characteristics, expressed as frequencies and percentages for categorical variables, and as mean ± standard deviation (SD) or median (interquartile range, IQR) for continuous variables. Group comparisons were made using the Chi-square test or Fisher’s exact test for categorical variables, and the independent sample *t*-test or Mann–Whitney U test for continuous variables, depending on data distribution.

Survival analyses were conducted using the Kaplan–Meier method, and differences between groups were evaluated by the log-rank test. Overall survival (OS) was defined as the time from diagnosis to death from any cause, while progression-free survival (PFS) was defined as the time from diagnosis to either disease progression or death. Cox proportional hazards regression analyses were performed to identify prognostic factors. In univariate analyses, the effects of individual variables on survival were assessed. Variables with statistical significance were subsequently included in the multivariate Cox regression model using a backward selection approach. Results were presented as hazard ratios (HRs) with 95% confidence intervals (CIs). To determine the discriminative ability of NPS for survival prediction, receiver operating characteristic (ROC) analysis was conducted, and the area under the curve (AUC) was calculated. The optimal NPS cutoff value was determined using the Youden Index.

Whenever feasible, molecular profiles were gathered, in addition to laboratory and clinical data. In particular, information about mutations in the epidermal growth factor receptor (EGFR) and the anaplastic lymphoma kinase (ALK) rearrangement was taken from the electronic database. Molecular data were available for 140 patients (56%) of the study population. Five patients (3.6%) had ALK rearrangements, while 25 subjects (17.9% of the cohort under study) had EGFR mutations. These genetic changes were excluded from the survival analysis due to the lack of data on the complete cohort; however, their proportions aligned with known data on the epidemiology of non-small cell lung cancer.

## 3. Results

A total of 250 patients with NSCLC were included, with a mean age of 64.3 ± 9.2 years. Of these, 58% were male and 42% were female. Regarding smoking status, 40% were current smokers, 35% were former smokers, and 25% had never smoked. Based on the Naples Prognostic Score (NPS), 135 patients were classified as low NPS (≤2) and 115 as high NPS (>2). No statistically significant differences were observed between the NPS groups in terms of age, gender, smoking status, or TNM stage (*p* > 0.05). However, serum albumin levels were significantly lower, and both NLR and LMR were significantly higher in the high NPS group (*p* < 0.001). Detailed demographic and clinical characteristics are summarized in Table 1.

**Figure 1:**
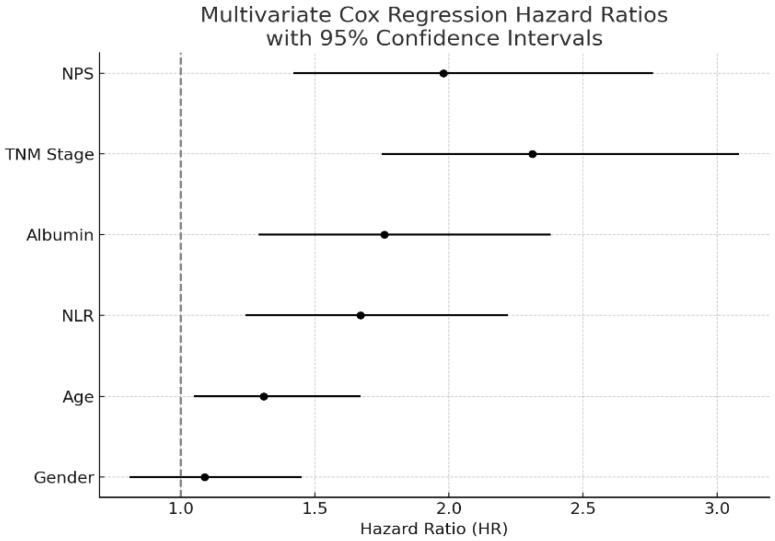
Forest plot illustrating the results of the multivariate Cox proportional hazards regression analysis for overall survival in patients with non-small cell lung cancer (NSCLC). The model includes the Naples Prognostic Score (NPS), TNM stage, serum albumin level, neutrophil-to-lymphocyte ratio (NLR), age, and gender. Hazard ratios (HRs) are presented along with 95% confidence intervals. NPS, TNM stage, and albumin were identified as independent prognostic factors.

In the univariate Cox regression analysis, high NPS (>2) was associated with significantly reduced survival (HR: 2.11, 95% CI: 1.55–2.88, *p* < 0.001), along with advanced TNM stage (HR: 2.47, 95% CI: 1.83–3.32, *p* < 0.001), low serum albumin level (HR: 1.89, 95% CI: 1.42–2.54, *p* < 0.001), and high NLR (HR: 1.67, 95% CI: 1.24–2.22, *p* = 0.002). Gender was included in the multivariate Cox regression analysis; however, it was not found to be an independent prognostic factor (HR: 1.09, 95% CI: 0.81–1.45, *p* = 0.58). In the multivariate Cox regression analysis, NPS remained an independent prognostic factor (HR: 1.98, 95% CI: 1.42–2.76, *p* < 0.001), as did TNM stage (HR: 2.31, 95% CI: 1.75–3.08, *p* < 0.001) and serum albumin level (HR: 1.76, 95% CI: 1.29–2.38, *p* = 0.001). The results of the regression analyses are presented in Table 2 and visualized in Figure 1.

**Figure 2:**
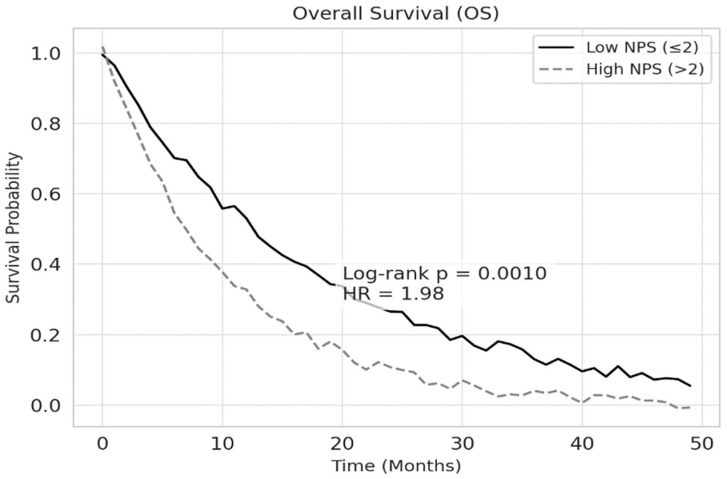
Overall survival according to the NPS in patients with NSCLC. Patients with high NPS (>2) demonstrated significantly shorter OS compared to those with low NPS (≤2) (log-rank *p* = 0.001). The HR of 1.98 indicates an almost twofold increased risk of death in the high NPS group. The 1-year OS rate was significantly higher in the low NPS group (72.4%) compared to the high NPS group (44.3%).

**Figure 3:**
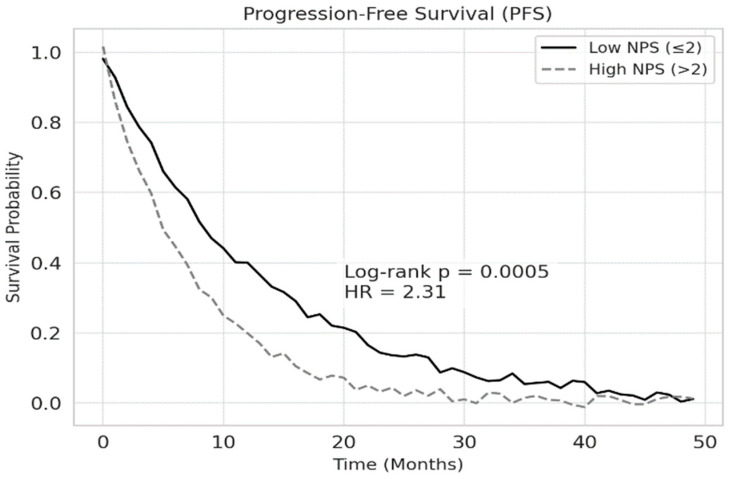
Association between NPS and PFS in patients with NSCLC. Patients with high NPS (>2) had significantly shorter PFS compared to those with low NPS (≤2) (log-rank *p* = 0.0005). A hazard ratio (HR) of 2.31 indicates that high NPS is associated with more than a twofold increased risk of disease progression.

According to the Kaplan–Meier survival analysis, the median overall survival (OS) was 18.2 months in the low NPS group and 10.4 months in the high NPS group (*p* < 0.001). Similarly, median progression-free survival (PFS) was 12.5 months for patients with low NPS and 7.3 months for those with high NPS (*p* < 0.001). The 1-year overall survival rate was 72.4% in the low NPS group and 44.3% in the high NPS group. These findings are illustrated in Figure 2 and Figure 3, respectively.

**Figure 4:**
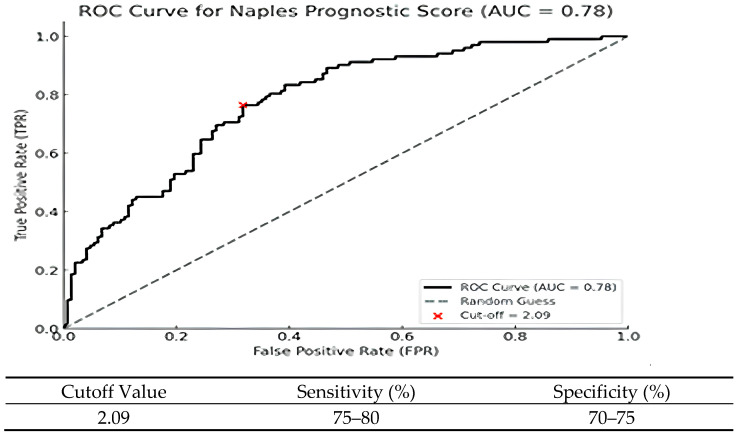
This ROC curve evaluates the ability of the NPS to predict survival outcomes. The AUC value was 0.78, indicating good discrimination. The best cutoff point determined using the Youden Index was 2.09, which provides a balance between sensitivity and specificity. A higher NPS value was associated with worse survival outcomes, supporting the prognostic importance of the NPS in patients with NSCLC.

Receiver operating characteristic (ROC) analysis revealed that NPS had good prognostic performance for survival, with an AUC of 0.78 (95% CI: 0.72–0.84). The optimal cutoff value determined by the Youden Index was 2.09, providing a sensitivity of 75–80% and specificity of 70–75%. The ROC curve was plotted using 1-year overall survival (death within one year) as the endpoint. These findings are shown in Figure 4.

In subgroup analyses, high NPS was significantly associated with reduced survival across different clinical categories. This included histological subtypes (adenocarcinoma and squamous cell carcinoma, *p* < 0.01), TNM stages (with a more pronounced effect in stage III–IV, *p* < 0.001), smoking status (*p* = 0.002), nutritional status based on albumin levels (*p* < 0.001), and age groups (greater effect in patients >65 years, *p* < 0.01).

## 4. Discussion

Establishing a reliable prognostic prediction system is of great importance for risk stratification and planning appropriate treatment strategies in patients with non-small cell lung cancer (NSCLC). The assessment of systemic inflammation and nutritional status is particularly valuable in evaluating their impact on clinical outcomes. The Naples Prognostic Score (NPS), a novel scoring system that reflects both the inflammatory and nutritional status of the patient, has been shown in previous studies to predict outcomes with greater accuracy compared to other prognostic scoring systems [21]. This study is a retrospective analysis that evaluates the prognostic value of the NPS in patients with NSCLC. Our study aims to comprehensively investigate the impact of this prognostic score, which incorporates markers of inflammation and nutritional status, on overall survival and to demonstrate its predictive utility across different patient subgroups through subgroup analyses. Our findings indicate that a high NPS is significantly associated with poorer survival outcomes in NSCLC patients and suggest that NPS may serve as an independent prognostic factor.

The prognostic role of inflammatory and nutritional parameters is gaining increasing importance in cancer patients. Considering the simplicity of the NPS calculation and the consistency of standards across most studies, our study serves as one of the guiding investigations into the potential utility of the NPS in prognostic assessment among patients with NSCLC [22,23].

Parameters such as NLR, LMR, albumin, and total cholesterol have individually been evaluated in various malignancies and shown to be associated with survival. In a study conducted by Chen and colleagues, survival outcomes in patients with obstructive colorectal cancer were evaluated, and it was shown that changes in immune and inflammatory indicators, such as NLR and LMR, could serve as a strong reference for prognosis prediction [24]. Nutritional indices such as the Prognostic Nutrition Index (PNI), Nutrition Risk Index (NRI), and Controlling Nutritional Status (CONUT) are recognized as independent risk factors affecting OS in cancer patients. The NPS, by integrating all these parameters into a comprehensive assessment of inflammation, malnutrition, and survival status, demonstrates superior predictive performance compared to PNI, NRI, and CONUT scores [25]. In a study conducted by Junhong and colleagues on a group of patients newly diagnosed with glioblastoma multiforme, it was found that the NPS is an independent prognostic indicator in newly diagnosed GBM patients and that the prognostic ability of the NPS is superior to that of the CONUT score [26].

However, establishing fixed and optimal predictive cutoff values for continuous variables across different studies remains a significant challenge. This limitation complicates the general applicability of such parameters and hinders standardization in clinical practice [27]. In particular, the role of inflammation in cancer progression has emerged as a critical area of research, especially when evaluated in the context of the immune response and its interaction with the tumor microenvironment. Previous studies have demonstrated that an elevated systemic inflammatory response is associated with poor prognosis, while nutritional deficiencies negatively impact survival [28,29]. Moreover, high NPS levels have been significantly associated with decreased survival outcomes in various malignancies such as colorectal cancer, endometrial cancer, and pancreatic cancer [30,31,32]. When the current literature is reviewed, it is observed that studies on the prognostic value of the NPS have mostly focused on gastrointestinal cancers such as gastric, colorectal, and hepatocellular cancers. For example, Gennaro et al. showed that a high NPS negatively affected survival in gastric cancer patients and should be considered an independent prognostic marker [33]. Similarly, Oing Li et al. have demonstrated that a high NPS is associated with poor prognosis in endometrial carcinoma [32]. However, studies conducted near NSCLC are limited, and this study is one of the rare ones highlighting the prognostic power of the NPS in NSCLC patients. Our study demonstrated that high NPS values were significantly associated with poorer OS and PFS. According to the results of the ROC analysis, the Naples Prognostic Score (NPS) demonstrated high performance in predicting survival (AUC: 0.78). The optimal cutoff value identified (2.09), with adequate sensitivity (75–80%) and specificity (70–75%), suggests that the NPS may serve as a suitable classification tool for clinical use. These findings suggest that the NPS may serve as a strong prognostic indicator in patients with NSCLC.

Our subgroup analyses revealed that the prognostic value of the NPS may differ across specific patient subgroups. In particular, in the multivariate Cox regression analysis, the NPS remained an independent prognostic factor, along with TNM stage and serum albumin level. Subgroup analyses revealed that the negative impact of a high NPS on survival was particularly more pronounced in patients with advanced-stage (Stage III–IV) NSCLC. This finding suggests that in the later stages of NSCLC, the impact of systemic inflammation and nutritional deficiencies becomes more pronounced and clinically relevant. In advanced cancer, the increased tumor burden and metabolic demand may exacerbate inflammatory responses and contribute to cancer-related cachexia, hypoalbuminemia, and lipid metabolism disturbances. These factors are known to negatively affect immune competence, treatment tolerance, and overall prognosis. Although there are a limited number of studies in the literature investigating the prognostic value of the NPS in patients with NSCLC, the existing findings are consistent with our results. For example, a study by Guo et al. demonstrated that the NPS is an independent predictor of survival in patients with stage III NSCLC and that a high NPS is associated with poor prognosis [21]. Similarly, Zou et al. observed that the NPS is an independent predictor of survival outcomes in patients with locally advanced NSCLC who underwent resection following neoadjuvant therapy [11]. While most of these studies were conducted on patient populations with limited disease stages, our study performed subgroup analyses in a broader patient cohort and showed that the NPS is not only a general prognostic indicator but also a dynamic marker that reflects stage-dependent risk variation.

Furthermore, the type of treatment received may have an impact on the prognostic influence of nutrition and systemic inflammation. The possible impact of treatment modalities on survival outcomes is an additional factor to take into account. It is possible that therapies like immunotherapy or targeted agents may have introduced survival bias, especially in patients who were at an advanced stage, even though comprehensive treatment data were not systematically recorded in this study. It is becoming more widely acknowledged that inflammation and nutritional status can affect how well immune checkpoint inhibitors work [34]. In this situation, the NPS may function as a predictive biomarker for patients undergoing immunotherapy, in addition to being a prognostic indicator [35]. As researchers continue to explore the complex interplay between these factors, it will be essential to integrate comprehensive data collection methods in future studies. This approach could enhance our understanding of patient responses and ultimately improve treatment outcomes for those receiving immunotherapy. This idea warrants further investigation in cohorts explicitly treated with immune-based or targeted methods, where the link between systemic inflammation and treatment response may be more precisely assessed.

Additionally, other indices based on nutrition and inflammation, such as the Systemic Inflammation Score (SIS), the Prognostic Nutritional Index (PNI), and the Controlling Nutritional Status (CONUT) score, have been proposed as prognostic tools in a variety of malignancies, in addition to the Naples Prognostic Score (NPS). Serum albumin levels and peripheral lymphocyte count are the main indicators of immunonutritional status in PNI, whereas albumin, total cholesterol, and lymphocyte count are all included in CONUT to evaluate immunological and nutritional function. In contrast, SIS uses serum albumin and the lymphocyte-to-monocyte ratio to calculate systemic inflammation. As determined by net reclassification improvement analysis, a recent study found that the CONUT score outperformed the PNI in prognostic discrimination when it came to predicting long-term mortality in patients with acute coronary syndromes [36]. The NPS incorporates a wider range of inflammatory and nutritional parameters and may provide a more thorough prognostic assessment, even though direct comparisons across tumor types are still scarce. This implies that, in terms of predictive accuracy, NPS may perform better than other individual scores like PNI, CONUT, and SIS. To confirm the relative prognostic value of these indices in NSCLC and other cancers, however, prospective, multicenter studies are necessary.

There is currently no specific study that directly investigates the prognostic value of the NPS in patients with metastatic NSCLC. However, some studies involving NSCLC patients have evaluated the overall impact of the NPS on survival. For example, Elia et al. conducted a retrospective propensity score-matched study in surgically treated NSCLC patients and demonstrated that the NPS was significantly associated with overall and cancer-related survival. Their findings validated the NPS as an independent and practical prognostic indicator in operable NSCLC patients [23]. This observation supports the notion that the NPS may serve not only as a general prognostic indicator but also as a dynamic marker reflecting the evolving host–tumor interaction, particularly in advanced-stage disease. Nevertheless, that study did not specifically focus on metastatic cases. Therefore, further research is needed to determine the prognostic significance of NPS in metastatic NSCLC patients. In our study, 60% of the patient population consisted of advanced-stage (Stage III–IV) and 40% of early-stage (Stage I–II) NSCLC patients. This distribution provides an important opportunity to assess how inflammation and nutritional parameters vary with disease stage and how sensitively the NPS reflects these changes. In advanced-stage NSCLC, increased tumor burden, heightened systemic inflammatory response, and more profound nutritional deficiencies are more likely to occur. Accordingly, the NPS, which reflects both inflammatory and nutritional status, is expected to serve as a stronger prognostic indicator in advanced-stage patients.

In our study, the prognostic significance of the NPS was found to be significant in both adenocarcinoma and squamous cell carcinoma subtypes, suggesting that the NPS may serve as a universal prognostic indicator independent of histological subtype in NSCLC. NSCLC represents a highly heterogeneous group of diseases in terms of molecular profiles, treatment approaches, and biological behavior. However, systemic factors such as inflammation and nutritional status may influence disease progression regardless of histological subtype [37]. In the literature, the NPS has mostly been studied in gastrointestinal cancers. However, a limited number of recent studies have indicated that the NPS may also have prognostic value in NSCLC. For instance, Peng et al. demonstrated that the NPS is a significant predictor of both overall survival and cancer-specific survival in surgically treated NSCLC patients [22]. Although that study did not perform subgroup analysis by histology, its findings support the general prognostic utility of the NPS in the broader NSCLC population. Similarly, Zou et al. reported that the NPS is an effective prognostic factor in patients with locally advanced NSCLC [11]. Nevertheless, these studies included limited analyses based on histological subtypes. In contrast, our study specifically evaluated adenocarcinoma and squamous cell carcinoma subtypes separately and found that the NPS exhibited similar prognostic strength in both groups.

The role of smoking in cancer progression through its impact on inflammatory responses and nutritional status has long been recognized. In our study, the significantly negative effect of high NPS values on survival suggests that a history of smoking may influence disease course through these mechanisms. Smoking increases systemic inflammatory burden via oxidative stress, chronic inflammation, and immune suppression; it also adversely affects nutritional parameters such as appetite loss, metabolic disturbances, and weight loss. For example, studies have shown that the function of alveolar macrophages is impaired in smokers, which weakens the immune response and increases susceptibility to infection and inflammation [38]. In a large cohort study of 5594 NSCLC patients, current and former smokers had significantly higher mortality compared to never smokers, with current smokers showing a 68% increased risk of death. Importantly, longer durations of smoking cessation prior to diagnosis were associated with improved overall survival, especially in early-stage patients, underscoring the prognostic relevance of detailed smoking history in NSCLC [39]. As the NPS reflects both inflammatory and nutritional status, it has the potential to comprehensively capture the biological changes associated with smoking. Therefore, the observed significant association between high NPS and shorter survival in NSCLC patients with a history of smoking in our study can be interpreted as a result of this biological process. This finding suggests that smoking history should be considered not only as a risk factor but also as a clinical variable to be incorporated into prognostic modeling.

In our study, the prognostic impact of the NPS was more pronounced in patients over the age of 65. This finding suggests that the systemic deterioration associated with aging may be more accurately reflected by the NPS. Aging is known to alter immune function, leading to a state of chronic, low-grade inflammation referred to as “inflamm-aging”, characterized by elevated levels of pro-inflammatory cytokines (e.g., IL-6, TNF-α) and a decline in anti-inflammatory mechanisms [40]. Additionally, age-related changes in nutritional status—such as decreased serum albumin and total cholesterol levels—may further influence prognosis [41].

As the NPS integrates key indicators of inflammation and nutrition (NLR, LMR, albumin, and total cholesterol), it may be particularly sensitive in detecting prognostic differences in older patients with NSCLC. While there are few studies that specifically examine the prognostic value of the NPS in elderly cancer patients, the broader literature supports the relevance of systemic inflammation and nutritional decline as important prognostic factors in older populations [42,43]. Therefore, our findings suggest that the NPS could serve as a valuable tool in the prognostic assessment of elderly NSCLC patients. Incorporating a comprehensive evaluation of inflammation and nutritional status into clinical practice may aid in treatment planning and improving outcome prediction in this vulnerable population.

This study has several limitations. Firstly, its retrospective and single-center design may affect the accuracy and generalizability of the findings. The presence of incomplete patient records may have negatively impacted data quality, and multicenter studies with larger populations are needed to validate the prognostic value of the NPS with stronger evidence. Inflammatory and nutritional parameters are dynamic and may fluctuate over time, particularly in cancer patients; thus, longitudinal studies are required to better assess the impact of these variables on prognosis. In our study, the NPS was not compared with other prognostic indices such as the Prognostic Nutritional Index (PNI), Controlling Nutritional Status (CONUT), and the Systemic Inflammation Score (SIS). This limits the ability to determine the relative clinical utility of the NPS in comparison to other scoring systems.

İn addition, this study has several notable strengths. It includes a relatively large cohort of 250 NSCLC patients and provides a comprehensive evaluation of the Naples Prognostic Score (NPS) across various subgroups, such as histological subtype, TNM stage, smoking status, and age. The use of both univariate and multivariate Cox regression analyses supports the independent prognostic value of the NPS. Moreover, the ROC analysis adds methodological strength by demonstrating good predictive performance and identifying an optimal cutoff. Importantly, this study is among the few to investigate the prognostic utility of the NPS specifically in NSCLC, contributing meaningful evidence to the literature.

## 5. Conclusions

In conclusion, our findings suggest that the NPS may serve as an independent prognostic marker in patients with non-small cell lung cancer. The routine assessment of the NPS could aid in personalized treatment planning, particularly in advanced-stage patients. A holistic approach that incorporates inflammatory and nutritional status into clinical decision-making may improve outcomes, and further prospective, multicenter studies are warranted to confirm these results and strengthen the role of the NPS in clinical practice.

## Figures and Tables

**Table 1:** The demographic data of the patients and subgroup analyses have been provided.

Characteristic	Value
Total Number of Patients	250
Mean Age (±SD)	64.3 ± 9.2 years
Gender Distribution	Male: 58% (145)|Female: 42% (105)
Smoking Status	Active Smoker: 40% (100)|Former Smoker: 35% (87)|Never Smoked: 25% (63)
Naples Prognostic Score (NPS) Distribution	Low (≤2): 135 patients|High (>2): 115 patients
TNM Stage	Early Stage (I–II): 40%|Advanced Stage (III–IV): 60%
Serum Albumin Level	Low NPS Group: Normal|High NPS Group: Significantly lower (*p* < 0.001)
Neutrophil-to-Lymphocyte Ratio (NLR)	Low NPS Group: Low|High NPS Group: High (*p* < 0.001)
Lymphocyte-to-Monocyte Ratio (LMR)	Low NPS Group: High|High NPS Group: Low (*p* < 0.001)

**Table 2:** Summary of the results of univariate and multivariate Cox regression analyses evaluating the prognostic factors affecting survival in patients with NSCLC.

Variable	Univariate Analysis (HR, 95% CI, *p*-Value)	Multivariate Analysis (HR, 95% CI, *p*-Value)
High NPS (>2)	2.11 (1.55–2.88, *p* < 0.001)	1.98 (1.42–2.76, *p* < 0.001)
Advanced TNM Stage (III–IV)	2.47 (1.83–3.32, *p* < 0.001)	2.31 (1.75–3.08, *p* < 0.001)
Low Serum Albumin (<3.5 g/dL)	1.89 (1.42–2.54, *p* < 0.001)	1.76 (1.29–2.38, *p* = 0.001)
High NLR (>3)	1.67 (1.24–2.22, *p* = 0.002)	Not Significant (*p* > 0.05)
Low LMR (<4)	1.52 (1.18–1.96, *p* = 0.004)	Not Significant (*p* > 0.05)
Smoking History (Active vs. Never Smoked)	1.45 (1.12–1.88, *p* = 0.008)	Not Significant (*p* > 0.05)
Age (>65 years)	1.31 (1.05–1.67, *p* = 0.015)	Not Significant (*p* > 0.05)
Gender (Male vs. Female)	1.12 (0.84–1.48, *p* = 0.42)	1.09 (0.81–1.45, *p* = 0.58)

## Data Availability

The datasets used and/or analyzed during the current study are available from the corresponding author on reasonable request.

## Abbreviations

Abbreviation	Definition
NSCLC	Non-small cell lung cancer
TNM	Tumor, Node, Metastasis (staging system)
NPS	Naples Prognostic Score
NLR	Neutrophil-to-lymphocyte ratio
LMR	Lymphocyte-to-monocyte ratio
OS	Overall survival
PFS	Progression-free survival
PNI	Prognostic Nutritional Index
NRI	Nutrition Risk Index
CONUT	Controlling Nutritional Status
SIS	Systemic Inflammation Score
AUC	Area under the curve
ROC	Receiver operating characteristic
HR	Hazard ratio
CI	Confidence interval
SD	Standard deviation
IQR	Interquartile range
GBM	Glioblastoma multiforme
IL-6	Interleukin-6
TNF-α	Tumor Necrosis Factor-alpha
